# Navigating in a value-driven practice: a study of a Dutch Recovery College as a learning, social, and organizational space

**DOI:** 10.3389/fpsyt.2025.1625779

**Published:** 2025-10-15

**Authors:** Marloes M. C. van Wezel, Christien Muusse, Jenny Boumans, Floris J. A. Scheerstra, Annelies Broos, Judith Lize, Kelly Leunen, Martijn P. M. Kole, Anthony (Ton) Verspoor, Dike van de Mheen, Hans Kroon

**Affiliations:** ^1^ Tranzo Scientific Center for Care and Wellbeing, School of Social and Behavioral Sciences, Tilburg University, Tilburg, Netherlands; ^2^ Department of Reintegration and Community Care, Trimbos Institute, Utrecht, Netherlands; ^3^ Enik Recovery College, Lister, Utrecht, Netherlands; ^4^ Cavallo Advies, Utrecht, Netherlands

**Keywords:** Recovery Colleges, peer support values, empowerment, free space, co-creation

## Abstract

**Introduction:**

Recovery Colleges (RCs) facilitate a peer-supported learning environment, co-created bottom-up for and by people with mental vulnerabilities. They explicitly aim to facilitate something different from traditional mental healthcare services, as their ideology is rooted in an emancipatory movement (with focus on peer support, empowerment, and personal recovery). RCs’ ideology comes with key peer support values such as equity, reciprocity, connectedness and empowerment. This study provides an experiential description of an RC practice, scrutinizing how peer support (PS) values are enacted and how partakers experience such value-driven practice.

**Methods:**

This study employs triangulation by combining twin-interviews, participatory observations (with auto-ethnographic elements), and (internal) documentation. All aspects of this study were co-created with experiential researchers who are RC partakers. 26 RC partakers were interviewed by a duo of an academic and an experiential researcher. Additionally, the first author conducted participatory observations over several years.

**Results:**

RC practice is described as a learning, social, and organizational space, each with their own physical and experiential elements. Our analysis showed that enacting PS values ultimately was about making or holding space, which was experienced as carrying both opportunities and challenges for recovery. We zoom in on challenges regarding collaborative learning, taking up and safeguarding space, and organizational growth.

**Discussion:**

Our findings highlight how RCs facilitate opportunities for recovery by fostering spaces for collaborative learning, mutual support and co-creation, while also revealing the fragility of these spaces. Experiences in RC practice are highly context- and person dependent. Navigating in such practice therefore requires continuous reflection and dialogue among all involved. To allow for such a culture to emerge and sustain, organizational free space should be safeguarded, minimizing constraints or interference from external parties.

## 1 Introduction

Recovery Colleges (RCs) facilitate a space where people with mental vulnerabilities can engage in their recovery journey in their own unique way (1, 2). By adopting an educational model rather than a therapeutical one, RCs explicitly aspire to deviate from traditional mental healthcare services (1, 3). To that end, they facilitate a peer-supported learning environment, where peers (i.e., people who have lived experience with mental vulnerabilities) meet as equals to support, inspire and learn from each other (3–5). A self-help curriculum forms the basis of RCs, offering recovery courses (e.g., Wellness Recovery Action Planning (6) and Honest, Open, Proud (7)) and workshops that are co-created with peers. Within RC offerings, partakers are invited to disentangle themselves from the role of ‘patient’ and transform into active ‘students’, learning what gives their life meaning, what they need to live it, and how they can increase their agency in life.

The origins of RCs can be traced back to an emancipatory user movement in psychiatry that gained momentum in the 1970s. A central theme in this movement was the advocacy for increased agency and empowerment of individuals with mental vulnerabilities. The axiom ‘Nothing about us, without us’ symbolized the movement’s call to reorganize power dynamics, acknowledging that individuals with mental vulnerabilities have a fundamental right to have control over their own lives. It criticized contemporary psychiatry as an over-medicalized sector in which excessive medication prescriptions, coercive and freedom-depriving methodologies, and unconventional treatments (such as electroshock therapy), were the status quo. In the 1990s, the movement claimed space for experiential knowledge, as pioneers such as Patricia Deegan (8), Judi Chamberlin (9), Mary O’Hagan (10) and Wilma Boevink (11, 12) wrote impactful accounts on their lived experiences with mental vulnerability and institutionalization in psychiatric services. Based on these first-person accounts, the concept of recovery was redefined as a learning process in which individuals actively (re)gain agency, hope, meaning and purpose, rather than passively wait for a linear improvement, transforming from ‘mentally ill’ to ‘mentally healthy’ (13, 14). As Boevink (11) formulated it:

“The psychiatric system is not able to cure nearly as often as some would like to believe. Waiting for this to happen keeps us submissive and passive. It is better to ask; ‘What are obstacles in my life, and how should I deal with them?’. [ … ] We are not psychiatric disorders with care needs – we are people with lives to be led.”

This study specifically scrutinizes an RC in the Netherlands, where the emancipatory movement was also well-established and impactful (15). For example, a client union was established in 1971 (16, 17) and consumer-run initiatives, such as runaway shelters, emerged in the 1980s (17, 18). Besides these consumer-run initiatives, the first Dutch RCs were founded in the early 21st century, inspired by the RC model rooted in the United States and the United Kingdom (1, 2, 19). In this light, the RC model is a concrete manifestation of the emancipatory recovery movement, as it centers experiential knowledge and frames recovery as a personal learning process by facilitating peer recovery education.

Following from RCs’ roots in this emancipatory movement, peer support (PS) values such as equity, reciprocity, connectedness and empowerment are at the heart of their practices (20, 21). How these values are enacted seems varied across RCs. For example, in English RCs, all activities are co-produced by peers and mental healthcare providers, to aid a culture change within mental healthcare services (20, 22, 23). In contrast, RCs in the Netherlands are typically peer-run, meaning that co-creation among peers is more common than co-production with mental healthcare providers (although variance exists, too). Dutch RCs specifically aim to provide an autonomous alternative to mental healthcare services (24, 25). Their focus on bottom-up co-creation by and for peers contributes to diverse offerings, often reaching beyond a self-help curriculum, also facilitating informal peer-to-peer contact and volunteering opportunities (26).

Research on co-produced RC practices suggests positive impacts on students’ recovery and wellbeing (27–30) and cultural change within mental healthcare services (22, 31, 32). Remarkably, RCs that focus more on a peer-run approach, without involvement of mental healthcare providers, remain underexplored. Furthermore, qualitative investigations of RCs, while centralizing experiences, often focus on ‘intervention-like’ aspects such as characteristics and related outcomes or impacts (e.g., (4, 33)). Little attention is paid to how partakers experience and navigate in these value-driven RC practices, and possible challenges that could emerge. Namely, while existing literature seems ‘overwhelmingly positive’ (30), there are also signals that navigating in RC practice can be challenging (34). For example for clinicians experiencing the dynamic RC practice as challenging and uncomfortable (32) or for students experiencing staff not aligning with the RC’s ethos (30), a misfit with their peers (35) or imbalance in a group (36, 37). The possible challenging nature of RC practice is rarely addressed or elaborated on, especially in the context of peer-run RCs. To fill these gaps, this study investigates a specific Dutch RC adopting a peer-run philosophy, scrutinizing how PS values are enacted, how partakers experience such value-driven practice, and how partakers experience challenges that could emerge.

## 2 Methods

### 2.1 Study design

This qualitative study is part of a multifaceted, multi-year, preregistered research project (38). It employs triangulation of twin-interviews, participatory observations (with auto-ethnographic elements) and (internal) documentation, which is beneficial to acquire an in-depth comprehension of complex phenomena (39). During the study, close collaboration took place between academic researchers and experiential researchers (who are RC partakers) in the POP group^1^, integrating their experiential knowledge into study design, data collection, analysis, and dissemination of results (see (38) for details). This methodology aligns with an RC’s philosophy of co-creation and aims to enhance research quality (e.g., (40–42)). Ethical approval was granted by the university’s Ethical Review Board (#TSB_RP390). The study followed COREQ (43) and SRQR (44) guidelines.

### 2.2 Setting

This study was conducted at Enik Recovery College (‘Enik RC’ hereafter in short), which was established in 2015 as one of the pioneering RCs in the Netherlands. Enik RC is located in Utrecht, the country’s fourth largest city, and encompasses seven different locations within the region. While being hosted by an organization for sheltered and supported housing, the RC adopts a 100% peer-run philosophy, meaning that all partakers (visitors, students, volunteers and employees)^2^ have personal experiences with mental vulnerabilities. The offerings are diverse, ranging from an extensive peer-supported self-help curriculum to a social meeting point for informal peer-to-peer interactions. Volunteering options are plentiful, and Enik RC also hosts retreats, which are multi-day workshop series including overnight stays. All offerings are free of charge and no diagnoses or formal indications are required to partake. In 2023, Enik RC offered a total of 800 workshops and activities and 16 retreats, welcoming 1,300 unique partakers, and facilitating the work place of 50 employees and 200 volunteers (45).

### 2.3 Recruitment

Participants were recruited at Enik RC by means of flyers and word-of-mouth. Purposive sampling was used to recruit a sample representative of the Enik RC population, hence including partakers in all possible roles to ratio (e.g., the majority is visitor, student or volunteer, a minority is employee or has attended a retreat). Former partakers were also recruited via the network of Enik RC. Eligibility criteria are detailed elsewhere (38).

### 2.4 Participants

In total, 27 participants were recruited and 26 interviews were actually conducted (*M_age_=* 43.31 years, *SD=* 9.53, *Range=* 26 – 62). One participant could not be interviewed due to a crisis situation. Twenty participants identified as female, four as male, one as androgyn and one as non-binary. The sample encompassed a representative range of involvement durations (e.g., 42% with 1–2 years and 27% with 4+ years) and roles (e.g., 26% students, 20% volunteers, and 7% employees; see Table A.1, **Appendix A**).

### 2.5 Data collection

Participatory observations were conducted by the first author at Enik RC during team meetings, workshops and activities, volunteering, and social gatherings from November 2021 to February 2024. At that point, we concluded that the obtained data was sufficiently rich and profound [see (46)]. The first author mostly acted as participant-observer, except during team meetings where she took a more distanced observer role. Initially, observations were conducted open and unstructured, without the use of predefined topic lists or observation schemes. As data analysis evolved, observations were more guided by emerging themes. During participation, notes were taken on factual observations (e.g., way of working, how interactions evolved, content discussed), interpretations and personal reflections. These were later elaborated into field notes, with additional post-observation reflections included. All reflections were distinguished from observations by using square brackets and italics. The resulting database included field notes on informal conversations with employees (*n=* 9), team meetings (*n=* 16), workshop participation, including a four-day retreat (*n=* 19, 8 workshops) and informal conversations and observations within the context of the social meeting point or volunteering (*n=* 25).

Furthermore, internal documents of Enik RC, such as a vision document or guidelines for opening new locations (*n=* 11), and online materials, such as interviews published on the RC’s website (*n=* 6) were retrieved and included in the data analysis.

The twin-interviews were conducted in duos of one academic researcher and one experiential researcher (hence ‘twin-interviews’). Four experiential researchers were trained as interviewers over the course of three 3-hour training sessions, focusing on both theory and practical skills. All materials were collaboratively developed with experiential researchers. The twin-interviews were semi-structured, focusing on three areas: Background and Introduction (first encounter with Enik RC, involvement, expectations), Dynamics of Enik RC (experienced values and distinctiveness of Enik RC), and Personal Process (needs and perceived impact of Enik RC). Important values of Enik RC (reciprocity, equity, connectedness, empowerment and free space; emphasized by the RC manager and in internal documentation) formed the basis discussing the dynamics of RC practice. We refrained from presenting definitions of the values in the interviews, rather, their meaning was co-constructed during the dialogue, guided by participants’ experiences. Visual aids, including a summarized topic list and values card, were provided to participants for reference and clarity (see **Appendix B** for details). Interviews were conducted from August to October 2022. All interviews lasted approximately one hour and were audio-recorded, transcribed verbatim, pseudonymized and member-checked.

To provide readers with a vivid illustration of RC practice, photographs were taken and incorporated in collages when the manuscript was drafted. All individuals depicted in the photographs were not study participants and provided written consent for publication.

### 2.6 Data analysis

**Figure 1:**
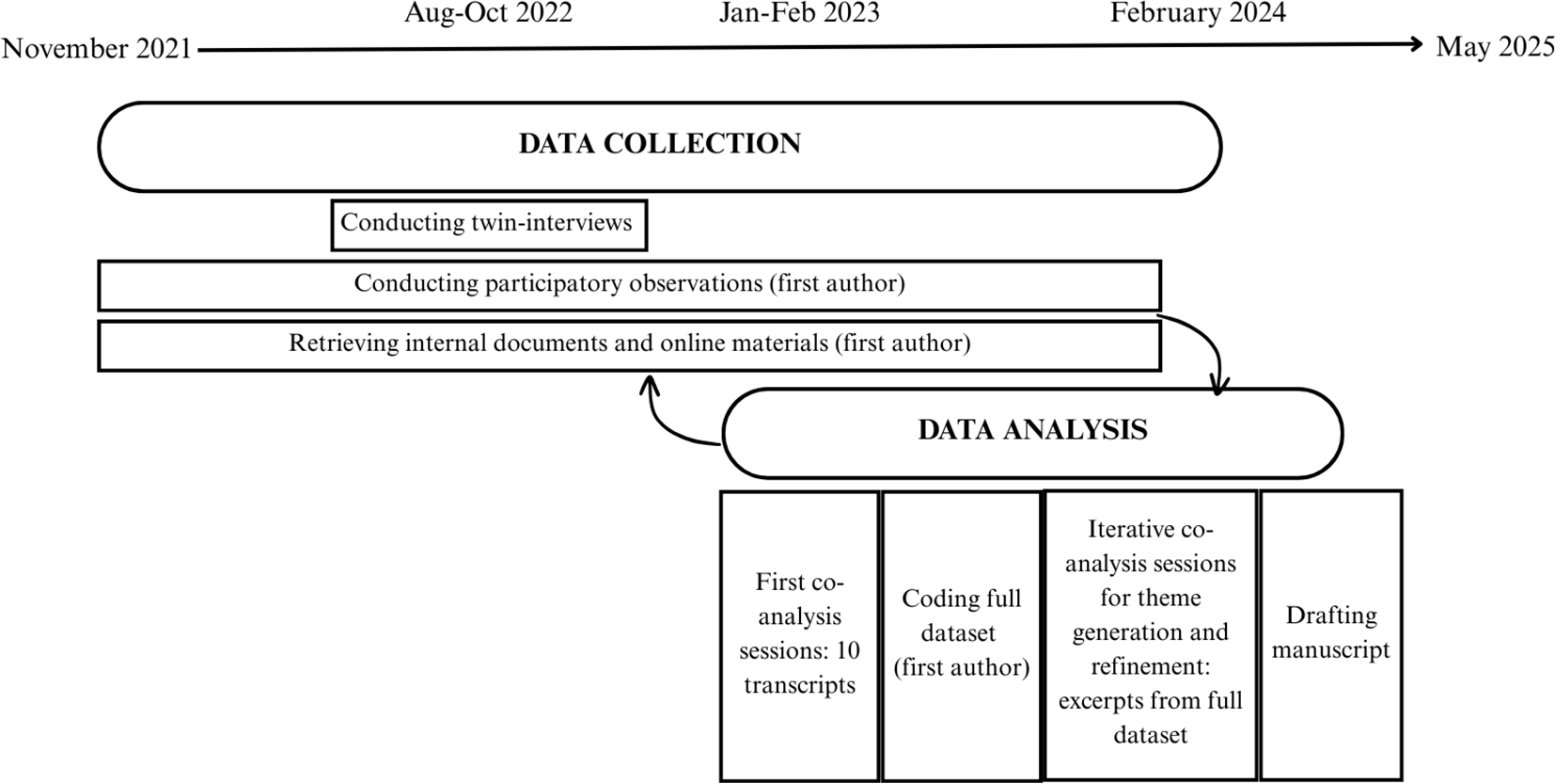
Diagram of data collection and analysis phases.

In co-research methods (such as participatory action research, PAR), it is important to leave enough space for attuning research methods to ideas, needs and abilities of the research group (47). Therefore, a predefined framework (e.g., adopting grounded theory as fixed method, as originally pre-registered (38)) was deemed less suitable. Adopting a flexible strategy allowed us to collaboratively analyze the data. (**Figure 1** provides an overview of data collection and analysis steps). Our analysis built upon several principles from constructivist grounded theory (48, 49) and reflexive thematic analysis (50, 51), namely being constructivist, interpretative and reflexive. Constructivism centers context-dependent meaning-making and embraces multiplicity of perspectives (48, 52). Furthermore, interpretative and reflexive analysis considers the researcher’s subjectivity as a resource rather than something to be minimized (50, 51), aligning with the co-research methodology that embraces diverse perspectives (40).

First, several co-analysis sessions were hosted in January – February 2023 to familiarize ourselves with the data, based on a subset of ten randomly selected interview transcripts. They were coded using open and inductive coding with Post -its, by addressing three questions: (1) What stands out? (2) What recovery-supportive factors are observed? (3) What recovery-impeding factors are observed? Our analytic process was dialogical as we jointly interpreted excerpts, discussed differing perspectives, and collaboratively decided on the most fitting codes. The emerging codes were clustered collaboratively into themes, with examples and initial identified patterns discussed. Subsequently, the first author applied these themes deductively to the remaining dataset using MAXQDA, while also inductively coding any new codes. New codes, themes and data fragments were discussed in further co-analysis sessions across several months, moving towards more latent coding, searching for meaning underlying the explicit concepts (51). At this point, an iterative, cyclic process of theme generation and refinement took place, as simultaneously additional observational data was collected (48). This additional data collection allowed for an in-depth scrutiny of identified themes, such as creating space, grounding our analysis further in the data. In total seven co-analysis meetings were hosted, though while (co-)writing this manuscript the analysis was shaped and finetuned further, sometimes with the experiential researchers, sometimes within the academic team. To illustrate, to collaboratively explore the central story line of our analysis, several experiential researchers wrote metaphorical accounts, for example describing RC practice as a musical jazz piece or West Coast Swing dance.

### 2.7 Reflexivity

In qualitative research, data collection and analysis are shaped by the researchers’ perspectives and judgments (53): what we see is in the eye of the beholder. The strength of co-creation in research methodology is inviting various beholders, each with their own backgrounds, experiences and beliefs (e.g., (40–42)). Our diverse research team included researchers contributing predominantly from an academic background (academic researchers) and researchers contributing predominantly from their lived and/or professional experiences at an RC (experiential researchers)^3^.

The first author, a female doctoral researcher with a background in Psychology (Bsc.) and Communication Sciences (Msc.), had limited professional knowledge of the mental health care sector at the onset of the study. However, while unfamiliar with RCs, she had personal experiences with recovery as an ongoing process in life, which likely influenced her observations and interpretations. The other academic researchers had extensive professional experience in recovery-oriented practice and community mental health research, shaping their pre-existing beliefs about the meaning and effectiveness of RCs.

At the project’s start, the experiential researchers group included employees, volunteers, and RC students with varying levels of involvement at Enik RC. Over time, this composition changed: some left, others joined, and some continued as experiential researchers even after ceasing active involvement at Enik RC. By the time of analysis and writing, several engaged experiential researchers were former volunteers or students. Their evolving relationship with Enik RC influenced their contributions, shaping both data collection and analysis.

Throughout the project, we consistently reflected on these diverse perspectives in team meetings and journal notes. The first author also documented personal reflections in field notes, distinguishing induced feelings and thoughts from direct observations. Recognizing the implications of our subjectivity, we embraced the richness of our team’s varied viewpoints. As one experiential researcher put it: “We reached a kind of consensus that moves beyond randomness towards objectivity – while acknowledging that absolute objectivity might not exist”.

## 3 Results

We present an analysis of how peer support (PS) values are enacted in RC practice in three ways: in the RC as a learning, social and organizational space. As we will show, in practice, these spaces overlap, and physical elements play a role in how they are given shape. For each space, we therefore first describe physical elements, through a scene-based narrative derived from the first author’s field notes, allowing the reader to imaginatively enter RC practice. Then, we describe how a specific space was experienced and which challenges emerged within that space.

### 3.1 The RC as a learning space

**Figure 2:**
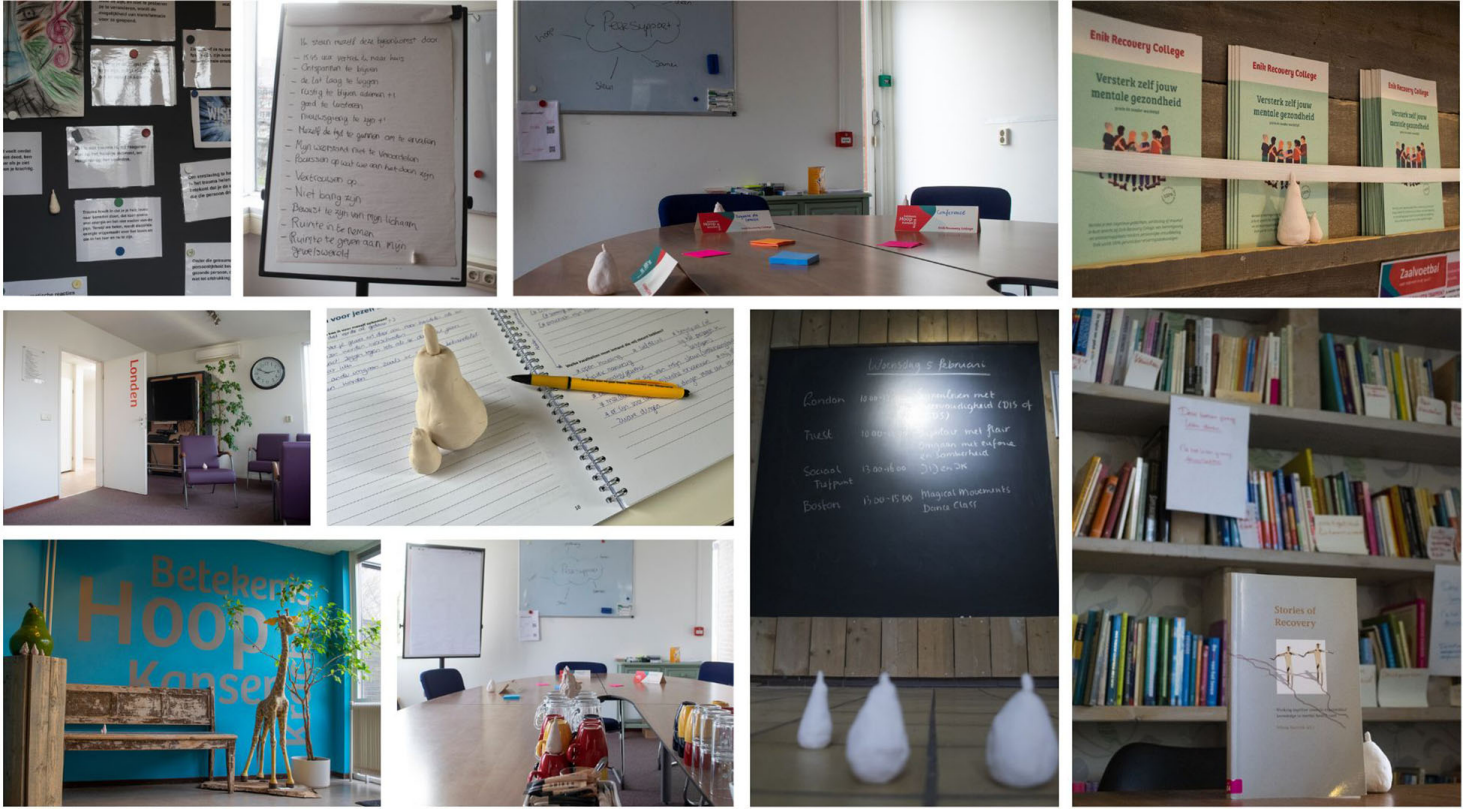
Collage of the learning space of RC practice.

Crossing the threshold into the RC practice with focus on the learning space, the first thing that stands out is the self-help program of the day, noted on a large chalkboard wall at the entrance. ‘Becoming your own loving parent’, ‘Exploring your boundaries’, ‘Coping with autism’ and ‘Am I on camera?’ are facilitated today. At the open reception desk, flyers detailing personal development opportunities are laid out and a library filled with books on recovery and experiential knowledge is available across the hallway. By going up the stairs, you encounter course room ‘London’, accompanied with an explanatory text. All course rooms have meaningful names, referring back to the roots of the emancipatory movement (e.g., O’Hagan, Trieste). The room is equipped with tools for collaborative learning, such as a digital and white board, flip-overs, and workbooks. A series of workshops has just finished, and the wall is covered with written flip-charts. A student points at the wall and remarks: “Look at how much we’ve done over the past weeks, how much insights we’ve acquired, how much we’ve learned”. This narrative illustrates physical elements of the learning space, which has been captured visually in a collage^4^ (**Figure 2**).

#### 3.1.1 Reflective dialogues through exchange of experiences

Partakers described the RC as a space for peer encounters in which sharing lived experiences of mental well-being, vulnerability, and recovery was essential. The data demonstrated that partakers experienced equity and reciprocity in social contact within the RC. Equity was often touched upon by interviewees in the sense that every experience was valued equally, with group facilitators participating on the same level as students, as a student mentioned: “What I also appreciate is that facilitators always share something about themselves in the introduction round. Then it is like, ‘Indeed, we are all equals here. We are here as human beings’” (Interview 10). Reciprocity, for interviewees, meant that everyone contributed to the exchange in their own way, by sharing experiences, offering comfort and understanding, or simply providing a listening ear. The RC therefore facilitated a value-driven space for exchange and dialogue, which seemed to inspire partakers and stimulate reflection, as one interviewee, involved as volunteer and student, explained: “If you come with a problem, some say, ‘I recognize that, I dealt with that this way’. [ … ] You can reflect on how this would work for you” (Interview 3). Therefore we label these interactions as ‘reflective dialogues’.

Our analysis indicated that reflective dialogues were important in how partakers experienced the RC as a learning space. More specifically, it was a collaborative learning space, in which ‘learning’ was an interactive process built upon equally valued experiential knowledge. During a workshop on the RC’s values, a facilitator explained what facilitating a collaborative learning environment entailed: “Without judgment or opinions. Not rigidly trying to teach someone the right way” (Field Note, Workshop Enik Values, 2022). The RC’s learning space in that way facilitated space for polyphony, where different viewpoints could co-exist and there were no fixed answers. In these collaborative learning processes, partakers learned more about themselves, their vulnerabilities and strengths, increasing self-understanding and self-compassion, because they gained a richer knowledge base or because others had a mirroring or inspiring exemplary function. The reflective dialogues facilitated space for a transformation from feeling helpless to feeling hopeful. During a ‘Talk about Recovery lunch’, a student shared:

“The lightness with which you share this gives me hope that the pieces of the puzzle will fall into place for me and others as well. I feel a heaviness, and I want to let it go. From you, I’m learning that it’s possible to bring lightness into it too” (Field Note, Talk about Recovery Lunch, 2023).

Collaboratively reflecting on recovery-related themes with peers also facilitated space to give meaning to own experiences. Hearing others’ stories and experiences implicitly supported partakers in making sense of their own. Though not always visible, this process underpinned many RC activities, particularly when partakers explored their own wants and needs. It opened up new ways of looking at things, inviting partakers to reevaluate how they want to relate to the world, others and themselves.

#### 3.1.2 Space to experiment

Besides a space for meaning-making, partakers also experienced the RC’s learning space as a space to experiment with (new) skills and roles. The RC provided various opportunities to explore roles such as co-creator and/or facilitator of workshops or volunteering (see ‘bottom-up co-creation’ in the section on organizational space). One way in which the RC aimed to facilitate this exploration was through ‘*Jij&Ik*’ (You&Me), scheduled moments in the social meeting points facilitated by ‘host peers’. An internal document described it as follows:

“*Jij&Ik* is a place to be, a place to share your story and listen to stories of others. [ … ] As a volunteer at *Jij&Ik* you contribute to the ambiance and core values that Enik wants to represent. [ … ] In particular, you are the point of contact for people who seek more information on Enik, its background, the program, and what you can get out of it. You can also help people who would like to volunteer. [ … ] You don’t know or master everything right away, of course. Within *Jij&Ik* you can learn what you need, and there is space to develop your own style” (Internal Document, 2023).

These meetings not only facilitated the exploration of pathways within the RC but also allowed host peers to learn from their role: “I never felt that I had something valuable to say. But in the role of host peer at *Jij&Ik*, [ … ] you suddenly realize: the other person appreciates it when I share my experience, how I solved something” (Interview 3).

Importantly, beyond practical roles and skills every partaker had the opportunity to experiment with the role of ‘expert of my own’, which related to meaning-making, too. Learning what experiences meant to them allowed partakers to make decisions that aligned with this meaning and what they wanted and needed in life. The value of empowerment was essential in this. As a student experienced:

“You are encouraged to rely more on your own strengths and responsibility. [ … ] Here, they assume a sense of wholeness, not necessarily focusing on illness [ … ] It is not merely the decision to go do something. It is also being able to choose within that. [ … ] Nobody says ‘You are a care avoider’, when I leave. They assume that I do what is good for *me*” (Interview 19).

An important tool to facilitate the adoption of this new role was the support document, assembled at the start of each meeting. The support document was considered “a tool designed to take responsibility for one’s own wellbeing” (Field Note, Workshop Enik Values, 2022). One facilitator explained: “You do it for yourself (what supports me), there may be contradictions in it, and everything can co-exist. [ … ] It is a replacement of group rules – you no longer depend on whether others follow the rules” (Field Note, Training ‘Learning to Facilitate’, 2023).

#### 3.1.3 Experienced challenges in the RC’s learning space

Above we described moments when partakers experienced PS values as supportive in the RC’s collaborative learning space. There were also moments when partakers experienced the value-driven RC practice as challenging to navigate in. One observed challenge in the RC’s learning space was that some partakers sought a more ‘traditional’ learning environment. Sometimes, they seemed more interested in retrieving information than in equal and reciprocal exchanges of experiences, as observed during a recovery workshop:

“I notice that the person next to me is very much looking for tips and tricks, advice and solutions. I sense in the group that that is not what Enik is intended for, they try to make that clear several times in different ways. ‘We don’t have a recipe book for you’. After a while, some people seem to become a bit annoyed by the recurring question about ‘the solution’. At some point, some laugh about it” (Field Note, Recovery Workshop, 2023).

This was reinforced by a former student, who shared having a need to learn from someone who can share their ‘expert knowledge’ sometimes:

“For example, I notice that when I am looking for an answer or a solution to things, it’s easily like, ‘You have to do that yourself’ [within the RC]. And that can also be empowerment sometimes [ … ] But for me, it sometimes feels as if you have to do everything yourself and as if you have to pull all the things you need out of thin air or something. Apart from – let me also mention reciprocity – that you share and exchange things. But for me that comes back to that equity: you exchange things, but you can never bring some kind of order to it that something is better than something else. [ … ] Sometimes you also just want, ‘How do I tackle the problem?’ Well, if someone is very good at that and has all the knowledge about it, then it is very useful if they share it” (Interview 24).

These examples show how the RC facilitated an open space for individual processes with no fixed answers, and that having to find your own pathway in that could induce tension for partakers seeking more guidance. This challenge of tolerating insecurity and facilitating openness within the RC was also discussed during a workshop on Enik RC’s values, as noted in a field note: “The facilitator tells us that sometimes facilitating is sitting on your hands, while you might believe you could help someone along. *[Personal reflection: I wonder whether it is always the right thing to strive for full equity here? Sometimes people can help one another with good advice, right? Or am I being naive]?*” (Field Note, Workshop Enik Values, 2022).

Another challenge in the RC’s learning space emerged in the dialogical space of group activities. Enik RC adopted a specific way of facilitating dialogical space, as was made explicit during a retreat, where each day started with a sharing:

“We start the sharing without guidelines. Someone shares a need for the day, to which another partaker responds empathetically, expressing a willingness to take it into account. Then, the facilitator explains that during a sharing, the intention is not to respond to one another. It is meant to create space for someone to express what is going on inside them. Once the person feels they have had enough space, they may close their sharing with ‘aho’, to which the group responds with ‘aho’ too, signifying something like ‘I have heard you’. Responding content-wise during a sharing may interrupt the person’s flow of thought or diminish the space they are given. The partaker is willing to give it a try “ (Field Note, Retreat, 2023).

While this sharing was retreat-specific, the RC’s group meetings were characterized by a similar open, non-judgmental atmosphere, where partakers mostly exchanged experiences and listened to each other. This could facilitate a fertile ground for an interactive conversation, while such conversation did not necessarily take place in the group context (but for example after a meeting or in the social meeting ground). The first author personally expected a ‘dialogue’ to be more profound and responsive within groups, and experienced another side to how the dialogical space was given shape, as described in this personal field note:

“When I say that I found the question confronting, I become emotional. The group responds very sweet, 90% non-verbal, just a friendly glance: little is said other than ‘thank you for sharing’. [ … ] *[Personal reflection: Later, in the car, driving back home, I feel pretty lonely. The theme of the meeting affected me, but to me, it [the exchange of experiences, red.] lacked depth, and I can’t discuss it with anyone now]*” (Field Note, Recovery Workshop, 2022).

In this example, the non-judgmental attitude of the group facilitated space for openness, but it also closed-off space for interactive, profound exchange, personally leading to feelings of safety but also loneliness. The examples illustrate how the dialogical space within the RC was not always about an interactive conversation, but also about opening up, making space for one’s story, being heard and listened to, and getting inspired by others. The context and needs of partakers seemed to impact how the dialogical space was given shape in practice.

#### 3.1.4 Zooming out: learning space within the RC

When zooming out, describing RC practice as a learning space showed how learning was considered a collaborative, dialogical process of experiential exchange among peers, and experimenting with (new) roles and skills. The learning space therefore had two layers: a relational one, where peers collaboratively learned, and an intrinsic, personal one, where partakers explored their own needs and abilities, reconnecting with themselves as experts. The RC held space for unique lived experiences, where partakers could (re)discover their sense of self, their identity, and their own meanings. The RC’s learning space was experienced as fostering positive learning experiences, and at times challenging to navigate in. Specifically, it seemed that this way of learning in an open, free space could evoke tension, which can also be inherent to learning processes. Finding one’s own pathway and tolerating openness and insecurity could be challenging, as became clear in the dilemma of whether or not to share expert knowledge, following from a particular understanding of equity. This analysis illustrates how making space – for self-expression, polyphony, meaning-making, not-knowing and knowing (as expert of your own) – was a recurrent theme within the learning space the RC aimed to facilitate.

### 3.2 The RC as a social space

**Figure 3:**
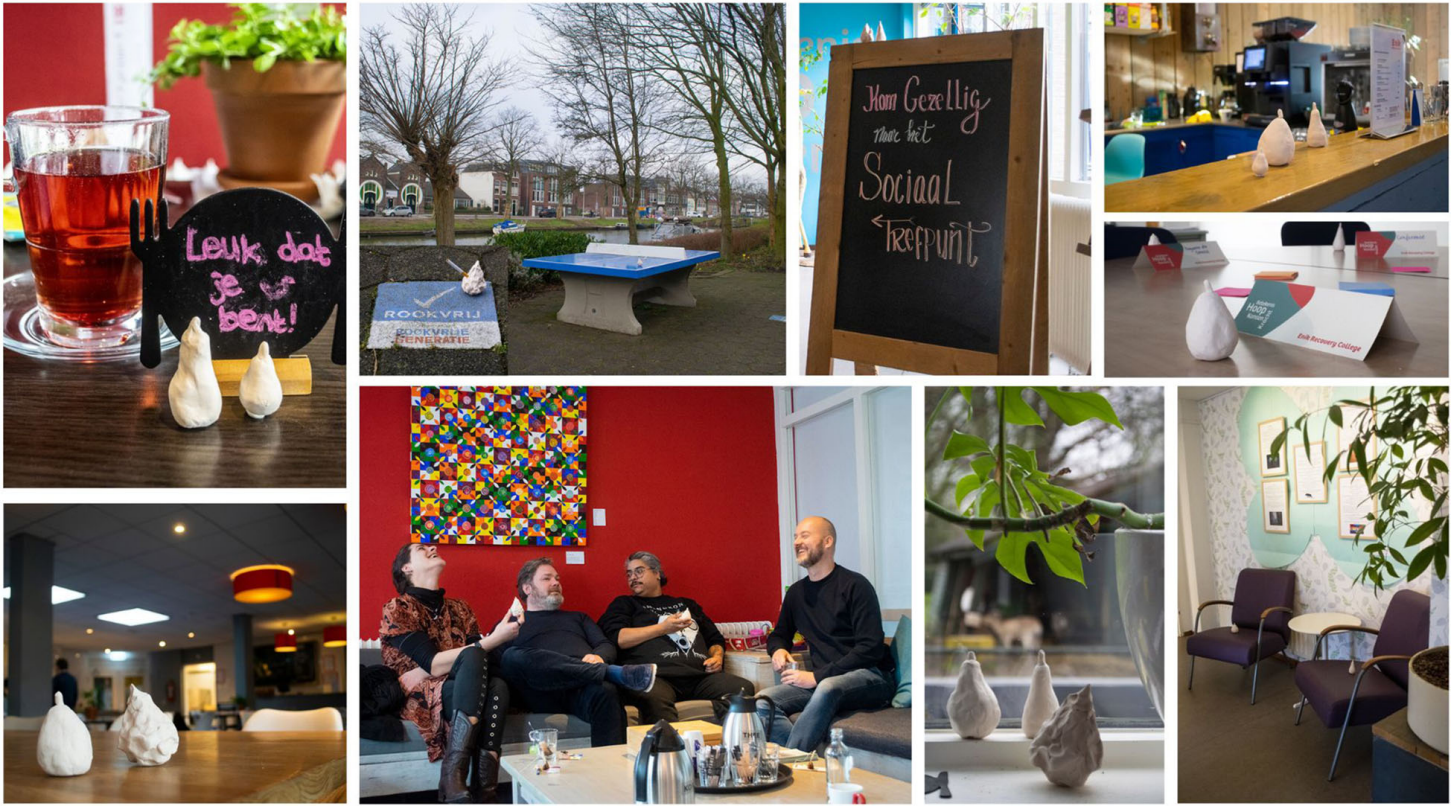
Collage of the social space of RC practice.

Stepping through the door into the RC with the focus on the RC as a social space, you encounter a wooden-framed chalkboard with colorful letters, inviting you in: “Come and join us at the social meeting point”. As you walk across the hallway you enter that social meeting point, with various seating arrangements to encourage social interaction and a menu with healthy, affordable meals. Outside you see a garden, including small sheds for goats and pigs. There is also a ping pong table: “I notice a group [partakers, red.] smoking cigarettes, gathered around a ping pong table covered with autumn leaves. It is obviously a place to meet, but not to play ping pong”. Stepping back inside, entering a course room, tea and coffee facilities are present at the table, and name signs are written, to get to know your fellow partakers. Throughout this scene you have encountered physical elements of the RC’s social space, again visualized in a collage (**Figure 3**).

#### 3.2.1 Sense of belonging

Besides providing learning opportunities, peer-to-peer interactions also had a strong social component. Our analysis revealed that sense of belonging was an important theme characterizing partakers’ experience in RC practice as a social space. In social encounters within the RC, connectedness was a recurring theme: “We are all here with a certain vulnerability, and being at Enik, we are also willing to open up, to dare to show it. I do feel a sense of connectedness in that” (Interview 16). Peers found recognition with one another, decreasing feelings of loneliness and ‘alienation’, as a former student explained:

“Of course, you know – also from doctors and stuff – that you are not the only one. But when you meet someone and talk to someone real life, and they experience similar pain or difficulties, you realize; ‘I really am not alone’. [ … ] It is more convincing in a way. On the one side, it is unpleasant that others suffer too, but on the other side it is nice to be able to talk about it with each other” (Interview 16).

Interviewees that had settled within the RC expressed a sense of belonging, oftentimes referring to the RC as a community they felt connected to. Some interviewees even referred to their RC contacts as family. One interviewee shared a turning point, where people would greet them by name when entering the building. “It was such a great feeling. I felt, ‘Now I really belong’. And that is very important to me because I never really felt as if I belonged anywhere” (Interview 9).

#### 3.2.2 Social safety

Another important theme in the experienced social space of the RC was social safety. The data revealed that the RC was experienced as an accepting place ‘to be’, and many partakers expressed feeling acknowledged for who they were: “It really makes me feel like I can be myself, that I don’t have to hide, that I don’t have to pretend to be better, that I am who I am” (Interview 11). Here again, feelings of recognition and acknowledgment seemed the key, as described in the following field note:

“The fact that you are not alone is described as healing. And knowing that everyone has been through ‘shit’ (emotional baggage is considered a ‘stupid’ word) helps [ … ]. You share your vulnerabilities faster than elsewhere” (Field Note, Retreat, 2023).

A personal field note highlights the role of recognition and the accepting, non-judgmental atmosphere within the RC’s social space:

“After the meeting, someone reaches out to me especially, to share how they recognized what I said earlier. [ … ] She tells me how she always looks for the emergency exits in the cinema – something I do, but had not shared yet. *[Personal reflection: We find recognition with each other, rapidly. And none of this is crazy, weird or against the ‘norm’. During conversations this is often expressed; people like to come here at Enik, because here, anything can be discussed, with anyone, without judgment. ‘Simply, a cup of coffee and a chat’]*” (Field Note, Creative Workshop, 2022).

During an open mic, social safety was observed as an important characteristic of how partakers experienced RC practice:

“*[Personal reflection: What stood out to me during the open mic was that there seemed to be no reservations for those performing. While some found it nerve-racking, the space was safe enough for them to do it anyway. To be vulnerable. [ … ] You don’t need to have a beautiful voice to share a song that moves you. You don’t need to be a poet to recite a poem. The only thing that matters is being yourself and sharing what you want. I also heard people say that to each other – that it was so beautiful how everyone just dared to ‘be’]*” (Field Note, Open Mic, 2022).

Not only the accepting atmosphere of RC practice contributed to experienced social safety. Several partakers shared how they experienced and valued a ‘laissez-faire’ atmosphere within the RC, where people would not unwantedly interfere with each other’s process. One visitor metaphorically explained: “Within the RC, every partaker is engaged with their own garden, removing their own weeds, nourishing their own garden (i.e., self-help and self-love). [ … ] Because everyone stands in their own garden, it is very safe” (Interview 15). A host peer in the social meeting point also valued this from a support-provider perspective: “I show how I approach life, how I cope with things. But I am no longer trying to save the other. That is up to them. And I notice that this brings me so much peace” (Interview 17).

#### 3.2.3 Experienced challenges in the RC’s social space

As described, there were moments when partakers experienced PS values in the RC’s social space as helpful. On some occasions, navigating in this value-driven RC practice was challenging for partakers. For instance, taking up space was something that many partakers struggled with, and RC practice sometimes did not meet their expectations, as one interviewee explained:

“In principle, it is not intended for them [facilitators] to intervene if, for example, someone is very wordy. [ … ] Everywhere you go where there’s a group discussion, there’s usually a moderator who ensures everyone gets a chance to speak. That’s even the case in the government. And then, at Enik, where it’s supposed to be safe for everyone, and where I think more people come who find it difficult to take up space, that moderation is missing” (Interview 3).

The ‘principle’ the interviewee referred to concerned a specific understanding of empowerment. Beyond seeing empowerment as reclaiming control over one’s life and building self-confidence, a prevailing interpretation of empowerment within the RC strongly emphasized independence and avoidance of unwanted interference in that process (i.e., the experienced ‘laissez-faire’ atmosphere previously mentioned). This specific interpretation strongly relied on personal responsibility and was also conveyed in the training on how to facilitate:

“How to deal with this [people not being assertive] according to the facilitators; tolerate your own discomfort, do not intervene. There will be a point that one will stand up for themselves. It is their learning process. They will learn from experiences. I asked: ‘What if people drop out before reaching that point?’ To which the facilitator answered: ‘Sounds harsh, but then that is that. They will encounter this theme somewhere else, again’” (Field Note, Training ‘Learning to Facilitate’, 2023).

A former student actually stopped attending the RC due to this interpretation of empowerment in a boundary crossing situation, despite benefiting from the RC overall. This experience was not unique, as we heard about physical, sexual or verbal boundary crossing situations within the RC. At times, individuals felt well supported by peers (and importantly, not necessarily facilitators or employees) when this occurred. As taught in the training on how to facilitate:

“The conclusion seemed to be consistently: as facilitator, you are not responsible for the process of someone else. [ … ] However, there is another layer to facilitators: that of human being. As human being, you can respond. If you are affected by an argument as human being, you can articulate that. If you feel connected as human being with someone who walks away, you can follow them. But this applies to everyone in the group – not just the facilitators” (Field Note, Training ‘Learning to Facilitate’, 2023).

At other moments, however, it was challenging for individuals affected by these situations, for example because people believed they were not allowed to intervene. A former student suggested that the RC’s roots played a role in this and experienced it as recovery-undermining instead of supporting:

“To my understanding, Enik was founded by people coming from a situation where [ … ] they experienced very little self-direction, where decisions were made for them. [ … ] It is a reaction to people doing things for you. But recovery of always having to figure it out yourself and there is never anyone who does anything for you, requires someone to take on that role and show you, ‘You don’t have to do it all alone’. [ … ] To emphasize too strictly that everything has to come from yourself, is in fact failing to show people that they do not have to do it all alone” (Interview 24).

The interviewee encountered a simplified, individualized view of recovery in this situation, and emphasized the importance of collectivism and togetherness. The example highlights a tension: although collectivism was also an important pillar of the emancipatory movement, it risks being overshadowed by an overemphasis on individual agency and empowerment. The specific interpretation of empowerment equaling ‘not interfering’ could thus be in tension with the idea of the RC as a social place for connectedness and mutual support among peers. This was also illustrated by an employee experiencing a dilemma when partakers were absent. Based on this specific interpretation of empowerment, they believed that it was not allowed to contact absent partakers. However, this interpretation of empowerment could interfere with the experienced connectedness, as they explained:

“Now it’s like, if you don’t show up anymore, then you just don’t come anymore. We’re not going to call you saying we miss you or asking where you were. It’s your own responsibility, etc. And I think that sense of personal responsibility can often feel like, ‘Figure it all out on your own’” (Interview 13).

Several interviewees had experienced such kind of disconnection from their peers, despite previously perceiving connectedness.

#### 3.2.4 Zooming out: the RC as a social space

Taking a step back, describing RC practice as a social space showed how RC practice was often experienced as a peer community characterized by a sense of belonging and social safety. Again, this value-driven practice was experienced as both supportive and challenging at times, with one specific interpretation of empowerment as ‘no interference’ in the foreground. The process of mutual support within the RC’s social space seemed to revolve around making space for openness, authenticity and reciprocal acceptance, while navigating values as empowerment, connectedness and reciprocity.

### 3.3 The RC as an organizational space

**Figure 4:**
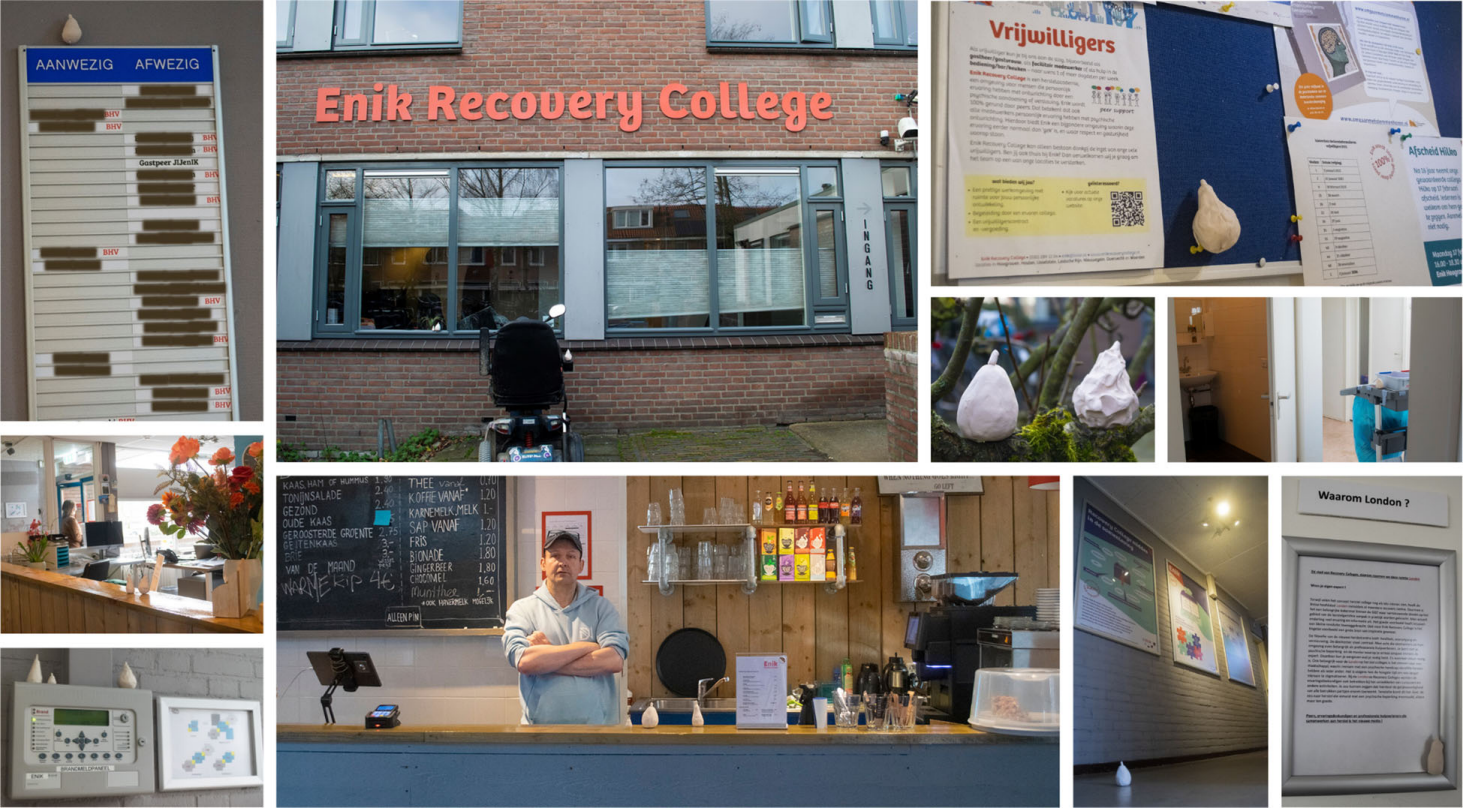
Collage of the organizational space of RC practice.

When approaching the RC practice as an organizational space you notice an office, visible through the windows. Walking in, you see the attendance board displaying names of working employees that day. As you wander through the building, you encounter various workspaces. At the reception desk – of which the glass partition has been removed to create openness – someone answers the phone. Large posters explaining the organizational structure of Enik as related to their host organization cover the hallway wall. You entered the RC during a lively moment, around lunchtime. In the social meeting point, a cook serves a grilled-vegetables sandwich, a bar tender a cup of coffee, and someone loads the dishwasher. Offices are spread across the building, in hallways and corners, where employees (behind closed doors) prepare their workshops or work on back-office tasks. Outside, individuals are gardening and feeding the animals. In a course room, two facilitators welcome a group. This scene illustrates physical elements of the RC’s organizational space, which has been visualized in a final collage (**Figure 4**).

#### 3.3.1 Low-threshold accessibility

A prevalent theme in how partakers experienced the RC’s organizational space was its aspired low-threshold accessibility. The RC aimed to facilitate a space that is “open and welcoming, easily accessible, and free of diagnoses” (Internal Document, 2019). Attendance in RC activities was always voluntarily, without referrals or indications. An interviewee explained how perceived unconditionality reduced thresholds to participate for them: “It’s not conditional in that sense. There’s no treatment relationship, no out-of-pocket costs, no questionnaires, [ … ] feeling pressured that it has to produce some kind of result, [ … ] or that I have to do it right” (Interview 18). Another interviewee highlighted the unlimited, open-ended nature contributing to low-threshold accessibility: “What I find a huge advantage of Enik compared to mental health care is that at Enik, you are not dismissed after a while. So that it’s not said, ‘We can’t offer you anything more’” (Interview 7).

The RC’s accessibility was also characterized by freedom to choose from a rich, available program. Within that, the RC held space for attuning participation to individual needs and aspirations, considering individuals as experts of their own. An employee explained:

“People come here because they want to, and not because they have to. [ … ] If you want to start in a group, but you find that difficult, you can start with something creative. Something with dance, music, meditation, or acting. [ … ] Plus, there are no waiting lists. I’ve met several people who were on waiting lists [for mental health care], and people get excited looking at the program together, ‘I’d like to do that’” (Interview 20).

Freedom of choice was also evident within RC activities, as exemplified in this field note of a creative workshop:

“Everybody receives some clay and with little introductions we start. Someone joins, they do not use clay but work in a color book. That’s all allowed. Later, someone leaves, and another comes from the social meeting point to check out what we are doing. The facilitator immediately asks whether they want to join. ‘I want to feel the clay with my hands, but I don’t feel like making anything’. That’s okay. Eventually, this person created something anyway (Field Note, Creative Workshop, 2022)”.

Beyond the program, the RC offered space to just ‘be’, for presence without obligations, for example in the social meeting point, library and outdoor areas.

Volunteers also described their working environment as accessible, emphasizing the RC’s flexibility. One volunteer for example explained how Enik RC helped them to start working again:

“They gave me space to learn at my own pace. Not like, you need to work 32 hours and if that doesn’t work out, after three times you are kicked out. Because that was not the case here, there was a lot less pressure” (Interview 8).

Within volunteering, there was also freedom to decide what to do, and what not. To illustrate, an interviewee explained that they did not feel comfortable turning on machines when having to leave. “I explain to them why this is the case, and there is much understanding for it. I don’t have to turn on the machine anymore. [ … ] At Enik, that is OK” (Interview 1).

#### 3.3.2 Bottom-up co-creation

A second theme of the experienced organizational space was bottom-up co-creation, as the RC aspired to organize all aspects of the RC by and for peers. One employee experienced that this contributed to the unique ‘spirit’ of the RC, that the organization really belonged to the peers themselves (Interview 9). The RC as organization largely depended on volunteers running the social meeting point, manning the reception desk and cleaning and maintaining the building and outdoor spaces. The self-help curriculum was also (partly) co-created. A volunteer for example shared how they designed and co-facilitated a workshop series and how empowerment was in play in that process:

“Being proud of myself. For example, that I took the initiative to come up with that series on [topic] and approached [name employee], saying, ‘Hey, I’d like to do a series on that’. Whereas I used to think, ‘People should come to me to ask me something, like, ‘Do you want this or that?’ or ‘Do you have something to say?’. But this time, I took the initiative myself” (Interview 3).

Importantly, the co-creative nature of the RC was not only ‘organized’ but also occurred spontaneously. For example, a volunteer in the social meeting point shared how they co-created the menu in interaction with peers:

“That just makes it nice that you have the freedom to do things according to your own judgment. Because of the feedback you get from the visitors, you sometimes ask them, ‘Would you like it if I made this or that?’ Then you get a tip or a suggestion. [ … ] ‘Next week or the week after, I’ll make it for you.’ And then the visitor comes back. It’s quite a nice interaction, you know” (Interview 8).

#### 3.3.3 Experienced challenges in the RC as organizational space

We saw that partakers experienced the RC’s organizational space as valuable, allowing them to engage in their own way. At the same time, this value-driven RC practice posed organizational challenges sometimes. Especially since the RC largely depended on volunteers in running their organization. The analysis made clear that the ideal to be accessible, with low thresholds and freedom to attune to individual needs, had another side to it. In volunteering, understaffing occurred, or volunteers felt that the workload was distributed unequally. A volunteer shared their feelings about a colleague who did not meet up to their expectations: “You are letting your colleagues down. You have a role, and with that role come certain tasks” (Interview 8). An employee responded to their concerns, explaining how the RC aims to facilitate freedom and space for unique recovery processes: “For some, merely being present is already an achievement”. This frustrated the volunteer, who felt it hurt team spirit: “Everyone else is just working really hard. Listen, either you participate, or you make sure you get a different role that suits you better” (Interview 8).

The ideal of bottom-up co-creation was also challenging in practice sometimes. Several interviewees felt there was little equity and empowerment in decision-making within the RC, as final decisions were made by employees. This becomes clear in the following example, where a volunteer experienced unwanted interference in how their co-created workshop was profiled, and described how that felt:

“For example with a workshop I facilitated, they changed the name. I thought, ‘It has to be our project, you stimulate empowerment, but then it has to be according to your rules up until the name. Then it is not my project anymore’” (Interview 14).

An employee (and former volunteer) shared their experiences with how the team struggled to include volunteers as equals in the organizational structures:

“There is a discrepancy between volunteers and employees [ … ] in the end, employees have an extra responsibility. [ … ] It makes perfect sense that this is necessary sometimes, that an employee rather than a volunteer is called when the alarm goes off at night for example. [ … ] But at team meetings for example, volunteers were not invited. [ … ] As volunteer that felt like, ‘I do the same work, I put my heart and soul into it, I do it well, I do it for little money. No salary, but a compensation. But I am not allowed to have a say. Where is the equity then?’” (Interview 13).

The topic repeatedly appeared on team meeting agendas across multiple locations as employees searched for a solution that has yet to be found. Inviting all volunteers to the team meetings was simply not feasible due to their large number.

Thus, a contributing factor that challenged the aspired values sometimes, seemed to be the RC’s scale. Followed the awarding of a tender, this increased, as it required scaling up and opening more locations. The expansion brought its own organizational challenges, as an employee explained:

“Growth means much more focus on organization, structure, and professional groups. Co-creation sometimes seems to fade away a bit or becomes too restricted. However, the growth resulting from the tender also brings pride: ‘We work hard, we see that it works, and that it is being recognized – we were jumping on the table in celebration!’” (Field Note, Informal Conversation with Employee, 2023).

The RC’s growth also enlarged the distance between the visionaries and experienced employees, and the work floor. Due to organizational restructuring, one of the RC’s founders was appointed as strategic advisor, reducing their direct involvement in the RC’s practice. Furthermore, the challenges we identified in our analysis were largely linked to uncertainty about the RC’s values and how to uphold them, which an employee suggested to be connected to the growth too:

“In the past, it was very dynamic and open, when partakers walked in at Enik they saw how things went. The contact was more personal. Now, it is no longer small-scale. [ … ] Previously, experienced workers were there to support those processes [when things were challenging, red.], but now, with the growth, that is becoming increasingly difficult. [ … ] The question now rises: do we still want this growth, or is this changing our values too much?” (Field Note, Informal Conversation with Employee, 2023).

#### 3.3.4 Zooming out: organizational space

All things considered, reflecting on the organizational space of RC practice revealed that partakers experienced low thresholds to participation and space to shape their involvement according to their own needs and abilities, stemming from the RC’s emphasis on recovery as a unique and personal process. Experienced unconditionality, open-endedness and flexibility of space contributed to this perceived low-threshold accessibility. Besides, the RC’s bottom-up co-creation contributed to personal learning experiences and to connection with the organization as a whole. It also opened-up space to translate ideas to practice, and co-create the organization with partakers. The organizational space also seemed essential in facilitating the other spaces (for learning and social encounters). The main challenge was guarding these value-driven spaces in a growing organization, where external pressures and the need for more capacity and professionalization sometimes put a strain on the enactment of the RC’s values. Here again, the process of making and holding space therefore seemed the crux in how the RC as organization was established and evolved.

## 4 Discussion

This study scrutinized an RC in the Netherlands, aiming to understand how PS values were enacted in practice and how partakers experienced this practice. Our analysis showed that enacting PS values ultimately was about making or holding space, which was experienced as carrying both opportunities and challenges for recovery. Three spatial aspects of Enik RC became apparent: a learning space, a social space and an organizational space. In all three spaces, the design of the physical space impacted how RC practice was given shape. The learning space of RC practice encompassed two layers. A first layer was relational and dialogical, where peers shared experiences and space was made for polyphony and a diversity of experiences. A second layer was more intrinsic within partakers, where they could explore their needs and abilities, (re)connecting with themselves as experts of their own. Together, the learning space was experienced to create space for new insights and meaning-making. In the RC’s social space, partakers could mutually support each other, find recognition and acknowledgement, and feel connected to the RC as a community. Finally, the RC’s organizational space emphasizing low-threshold accessibility and bottom-up co-creation could be experienced as facilitating freedom of choice, making way for partaker’s experiences and ideas to be implemented in practice. These findings resonate with previous qualitative evaluations that describe how partakers experienced mutual learning, connectedness, acknowledgment and empowerment within RC activities (e.g., (3, 29, 30, 33)). Notably, our analysis went beyond listing mechanisms of action as discrete elements. Recognized mechanisms in RC(like) contexts, such as facilitating a supportive, empowering environment, enabling reciprocal relationships and shifting power balance (4, 5, 54), were corroborated in our data, but additionally our analysis showed how they were interwoven, contextually enacted, and occurred across overlapping RC spaces. This deepens the understanding of RCs as dynamic practices where space for recovery is facilitated.

Besides the experienced opportunities of making space, the findings also illustrated how navigating in these RC spaces was dynamic and sometimes challenging. This supports earlier signals (32, 35–37) and answered to the call of Whish, Huckle (30) for more nuanced qualitative analyses of RC experiences. The variety of experiences within RC practice makes understandable how PS values are not pre-existing, independent constructs that merely need to be ‘translated’ into practice; rather, they are abstract concepts that gain meaning only through practice. Without actors that embodied the PS values, and specific contexts in which they were enacted, PS values remained empty concepts on a wall. Because of this person- and context dependency, the value-driven RC practice could be experienced as supporting recovery at times while undermining recovery at other times. In that line, the various spaces within RC practice could feel open and freeing, allowing partakers to feel empowered, learn new things and gain self-confidence, but they could also become pressured and closed-off, inducing feelings of insecurity, unsafety and invalidation. Our analysis suggests that experiences in RC practice were shaped by the way in which individuals navigated uncertainty, both in relation to their own recovery process and to the openness and flexibility of the RC context. We propose that two contextual conditions are important in this navigating process: (1) facilitating a culture of ongoing dialogue and reflection among all involved about how RC space is experienced, driven by curiosity and willingness to learn as a bottom-up co-created organization (see also (26)) and (2) safeguarding organizational free space to allow for such a culture with minimal interference from external parties. Achieving this requires awareness and dedication from both RCs and collaborating or related external stakeholders.

### 4.1 Relating observed dynamics to insights from adjacent fields

While (experienced challenges of) navigating in a value-driven practice received little attention in RC literature thus far, the observed dynamics can be related to concepts described in adjacent fields. For example, dialogical space is central to Peer-supported Open Dialogue (POD) and – while focused on individual support rather than a community context – its definition emphasizing space for flexibility and polyphony seems closely related to how RC practice was experienced:

For a dynamic to be dialogical, therefore, it must start without fixed objectives, within certain parameters, so as to allow for a free exchange that builds up layer by layer. [ … ] In addition, unlike the dialectical dynamic, there is no goal of a merging of viewpoints in order for a shared perspective to be reached. Each person can maintain their own perspective, and each perspective can hold more salience in particular circumstances – depending on the needs at the time (55).

POD views uncertainty as inherent to the experience of mental vulnerability. Tolerating it rather than rushing to solutions is considered essential to make space for individual processes to unfold, allowing individuals to recognize and articulate their own needs and experience agency (55, 56). This view was also evident in our observations of RC practice, where allowing space for individual meaning-making and recovery processes was central. However, we also observed that a narrow or simplistic interpretation of making space for individual processes – such as ‘not interfering’ or ‘no responding’ – could paradoxically reduce the experienced space intended to support recovery.

Moreover, the observed importance of creating space can be related to the concept of ‘free space’, which is considered the core value of expertise by experience in the Netherlands (57) and was identified as core value of Dutch RCs (26). In a brochure on RCs, Boertien and Harmsen (58) define its intrinsic aspect:

“Free space primarily refers to the inner free space a person can experience. Such experiences often mark a turning point in a recovery process. Despite the confinement and disruption caused by a ‘mental illness’, a person may experience a liberating moment – feeling a sense of breathing space, seeing light, feeling uplifted, or having a moment of empowerment, and so on “.

In the Dutch professional competence profile for experts by experience (i.e., a document outlining required skills, knowledge and behaviors in the profession), free space is described as a multi-layered concept, with intrinsic, relational and organizational aspects (57). On top of intrinsic free space, the authors emphasize space for personal experiences, meaning-making and multiple perspectives. In these accounts, in line with our observations, space to reflect on values and their meaning is considered conditional for designing value-driven practices such as RCs. As Boertien and Harmsen (58) put it: “Commitment to certain values requires continuously reflecting on their meaning for you, especially in challenging moments”.

The concept of free space also entails an organizational layer, as it advocates for an explicit shift in power dynamics, making space for influence of people with mental vulnerabilities (57). This can be related to the RC’s organizational space, where bottom-up co-creation aimed to make way for partaker’s impact on shaping RC practice. The implementation of this proved challenging at times, and although not elaboratively discussed, a factor of influence in this could be the RC’s positioning as part of a host organization for sheltered and supported living. The positioning of RCs is not straightforward because they want to facilitate an autonomous, alternative space, while also being embedded in the wider landscape of care and support (25). Organizing an RC therefore requires continuous balancing between ideals and values on the one hand, and system integration on the other hand (25, 59). Our findings showed that such balancing act could be challenging, especially when organizational growth is aspired or requested. These challenges are also experienced in the field of experiential knowledge and expertise within mental healthcare services, where persistent views and frameworks stemming from the traditional mental healthcare system hinder successful implementation (60, 61). Karbouniaris, van Gaalen (60) therefore state that a discretionary space and professional autonomy are required for experiential knowledge to be successfully embedded. In that line, a discretionary space is also essential for RCs to facilitate value-driven practices. Safeguarding the distinctiveness of RCs requires host organizations and partners to remain conscious of RCs’ foundational values and guiding principles, especially in the context of professionalization and scaling up.

### 4.2 Theoretical and practical implications

To truly understand experiences in RC practice, it is essential to realize that PS values only gain meaning in specific contexts. Partakers, in any role, continuously attune how to shape their involvement within the RC as a value-driven practice. This implication of our findings has both a theoretical and a practical dimension. Theoretically, it means that PS values are difficult to grasp in one standardized definition, as also illustrated by the varied definitions and conceptualizations of empowerment in the domains of recovery (62), the consumer movement (63) and social work (64, 65) (see also (66)). The question is whether we must aspire to achieve set-in-stone definitions of PS values without contextualization. To facilitate effective dialogue about what PS values mean, it is important to make explicit which definition is referred to, and why, explicating theoretical frameworks or ideologies underlying a specific definition (see also (58, 67)).

Practically, it means that a value-driven practice involves a continuous process of attunement and meaning-making, navigating individual needs and experiences of all involved, in the light of specific contexts. Navigating in such practice also means encountering challenges without fixed answer, requiring collaborative solutions. An open and free space allows this process to unfold, necessitating ongoing dialogue among all involved about what PS values mean in specific contexts and how an open free space can be safeguarded. Navigating in an RC practice therefore involves daring to ‘be with’ (or tolerate) uncertainty and discomfort at times, and collectively reflecting on that process. This not only requires engagement from the organization aiming to promote these values, but from everyone involved (partakers, but also related external stakeholders).

### 4.3 Strengths and limitations

The findings of this study should be interpreted in light of its strengths and limitations. The used methodology has several strengths, namely (1) triangulation of data sources across multiple years, (2) scrutiny of RC partakers beyond students alone, and (3) co-creation with experiential researchers. First, triangulation of interviews, observational data (with auto-ethnographic elements) and (internal) documents allowed for an in-depth scrutiny of RC practice (39, 68), especially since observational data was conducted over multiple years. Namely, frequently returning to a practice of study is important to obtain a profound understanding of (changing) dynamics in its social contexts (69). Second, this study contributes to the literature as one of the first to report on the experiences of RC partakers across all possible roles (visitor, student, volunteer, employee and former partakers). Existing RC research primarily focusses on students (e.g., (30)) or staff (e.g., (22)). However, a full comprehension of the dynamic, rich RC practice can only be achieved when considering the diversity and fluidity of roles and forms of engagement. As this study showed, the way PS values were experienced in practice depended on someone’s role or context. Third and finally, all phases of this study were conducted in collaboration with experiential researchers. This not only aligns with the RC’s philosophy of increased empowerment and valuing experiential knowledge, it also enhances the quality of the work (40–42). Co-research for example fosters an ongoing dialogue that aids to get disentangled from pre-existent beliefs or frameworks, stemming from academic backgrounds or personal experiences (40). Moreover, the combination of collaborating with experiential researchers and including auto-ethnographic accounts of the first author further strengthened the incorporation of experiential knowledge from different angles. The resulting database was therefore developed through deep immersion in practice, moving beyond mere observation.

These strengths come with limitations at the same time. For example, the study was conducted at one specific RC, while it is known that significant variance exists in how RCs are given shape, both within the Netherlands (26) and internationally (70). For example, dynamics within the RC may depend on the extent of its involvement with traditional mental healthcare services, funding or staff ratio. Even within Enik RC, its seven locations varied significantly, from small sized locations in a community center or library to a large sized self-managed location. At the same time, while RC practices may differ, we believe that the study’s implications apply to various RC practices or even PS groups more broadly, as they all share a foundation of approaching recovery as a collaborative learning process, with PS values as central pillars (26). Similar dynamics may even occur in other contexts that centralize values like equity and empowerment, aiming to break with traditions of therapeutic standards, such as in person-centered care and shared decision making (71), resource groups (72), POD (55), relational care based on the presence theory (73), or PAR (47). In that light, an intensive investigation of one specific RC could be an essential step towards profound understanding (74).

Finally, a caveat must be added to the co-research methodology. While we attempted to employ co-creation with experiential researchers in all research phases, this was not straightforward. Especially when writing up the manuscript, it was challenging to achieve a desired level of co-creation. One could say that co-research involves similar challenges as an RC practice: aiming for a low-threshold collaborative environment where values such as equity and empowerment flourish, yet also navigating within existent academic frameworks, responsibilities, habits and deadlines. Occasionally, researchers fell into the pitfall of traditional role divisions, such as academic researchers taking the lead and attempting to pre-structure working methods, and experiential researchers waiting for instructions (75). That said, the intensive collaboration in the pre-writing stages has significantly shaped this paper and supports the claim that this study adopted a co-research methodology. The potential for further improvement, particularly in the final stages, remains a critical consideration for co-research practitioners (76, 77).

### 4.4 Future directions

We could call for more empirical-analytic research on PS value-driven contexts exploring individual differences and contextual influences, to answer questions like “Who benefit from value-driven practices?” or “Which strategies help navigating in them?”. But we will not. Rather than advocating for additional research that aims to categorize these dynamics, we argue that a more meaningful direction lies in acknowledging their fluidity and situated nature. Any attempt to structure, categorize, or formalize these dynamics, even by qualitative inquiry (e.g., identifying patterns through thematic analysis), risks undermining this fluidity. Future efforts should therefore shift away from attempts at generalization and instead focus on bottom-up, co-created explorations, making way for polyphony and context-dependent meaning-making (for example, considering impact of organizational factors). Co-creative research methods such as PAR should be integrated as essential part of knowledge development, shifting power dynamics, increasing epistemic justice and improving research quality (see for example (78, 79)). Researchers, like partakers in value-driven practices, should embrace uncertainty and messiness (75, 80) and recognize that sometimes, there are no fixed answers.

## Data Availability

The datasets presented in this article are not readily available because of privacy protection. Requests to access the datasets should be directed to Marloes van Wezel, M.M.C.vanWezel@tilburguniversity.edu.

## Footnotes

1 POP stands for *Peer Onderzoekers Perspectief*, which translates to ‘Peer Researchers Perspective’ in English

2 RC partakers can be (and often are) active in multiple capacities within the RC: visitors are individuals who visit the social meeting point, students attend workshops or activities, volunteers work as co-creators and/or co-facilitators of the program or in hospitality, and employees are paid experts by experience facilitating RC offerings and working in back-office.

3 We write ‘predominantly’ because the distinction between academic and experiential researchers is not as black and white: academic researchers may have personal lived experiences, and experiential researchers may have academic knowledge and skills.

4 In the collages, the main characters are handcrafted clay pears that we co-created in the POP group. The fruit ‘pear’ translates to ‘peer’ in Dutch and is often used as a symbol for peer support.
