# Supplementary material for: Navigating in a value-driven practice: a study of a Dutch Recovery College as a learning, social, and organizational space
Source: Front Psychiatry. 2025 Oct 15;16:1625779. doi: 10.3389/fpsyt.2025.1625779 (PMC12570177; doi:10.3389/fpsyt.2025.1625779)
Supplement: Supplementary file 1 [file SupplementaryFile1.pdf]

Appendix A – Participant Descriptives

Table A.1

Age, Gender, RC Roles and Duration of RC Involvement of Interviewees (N= 26)

Id	Age	Gender	RC Roles	Involvement Duration (years)
1	60 – 69	Female	Volunteer	3 – 4
2	40 – 49	Female	Visitor	1 – 2
3	50 – 59	Female	Student, visitor, volunteer	3 – 4
4	30 – 39	Male	Volunteer	1 – 2
5	50 – 59	Female	Student, volunteer	1 – 2
6	50 – 59	Male	Student, visitor	3 – 4
7	40 – 49	Female	Student, volunteer	4+
8	30 – 39	Non-binary	Student, volunteer	1 – 2
9	50 – 59	Female	Employee	4+
10	30 – 39	Female	Student	1 – 2
11	40 – 49	Female	Student, visitor, volunteer, retreat	3 – 4
12	40 – 49	Female	Student, visitor	1 – 2
13	50 – 59	Female	Employee, student, visitor, retreat, former volunteer	4+
14	30 – 39	Female	Volunteer, visitor	1 – 2
15	50 – 59	Female/Androgyn	Visitor	3 – 4
16	40 – 49	Female	Former student	3 – 4
17	50 – 59	Female	Student, visitor, volunteer	1 – 2
18	40 – 49	Male	Employee, student	4+
19	50 – 59	Female	Student, retreat	4+
20	30 – 39	Female	Employee, student, retreat, former volunteer	1 – 2
21	30 – 39	Female	Student, volunteer, retreat	1 – 2
23	20 – 29	Female	Former student	3 – 4
24	30 – 39	Female	Former student, former visitor, former volunteer	1 – 2
25	20 – 29	Female	Former student, former volunteer, former retreat	3 – 4
26	30 – 39	Female	Former student	4+
27	40 – 49	Male	Former employee, former volunteer, former student	4+

Note. Interview 22 was not conducted due to a crisis situation.
