# Supplementary material for: Navigating in a value-driven practice: a study of a Dutch Recovery College as a learning, social, and organizational space
Source: Front Psychiatry. 2025 Oct 15;16:1625779. doi: 10.3389/fpsyt.2025.1625779 (PMC12570177; doi:10.3389/fpsyt.2025.1625779)
Supplement: Supplementary file 2 [file SupplementaryFile2.pdf]

Appendix B – Visual Aids Used in Twin-Interviews

Start interview

*First time Enik
*Involvement with Enik
*Expectations of Enik

Dynamics within Enik

*The core values of Enik
*Is Enik different from society at large?

Personal process

*Needs in own process
*Impact Enik on process

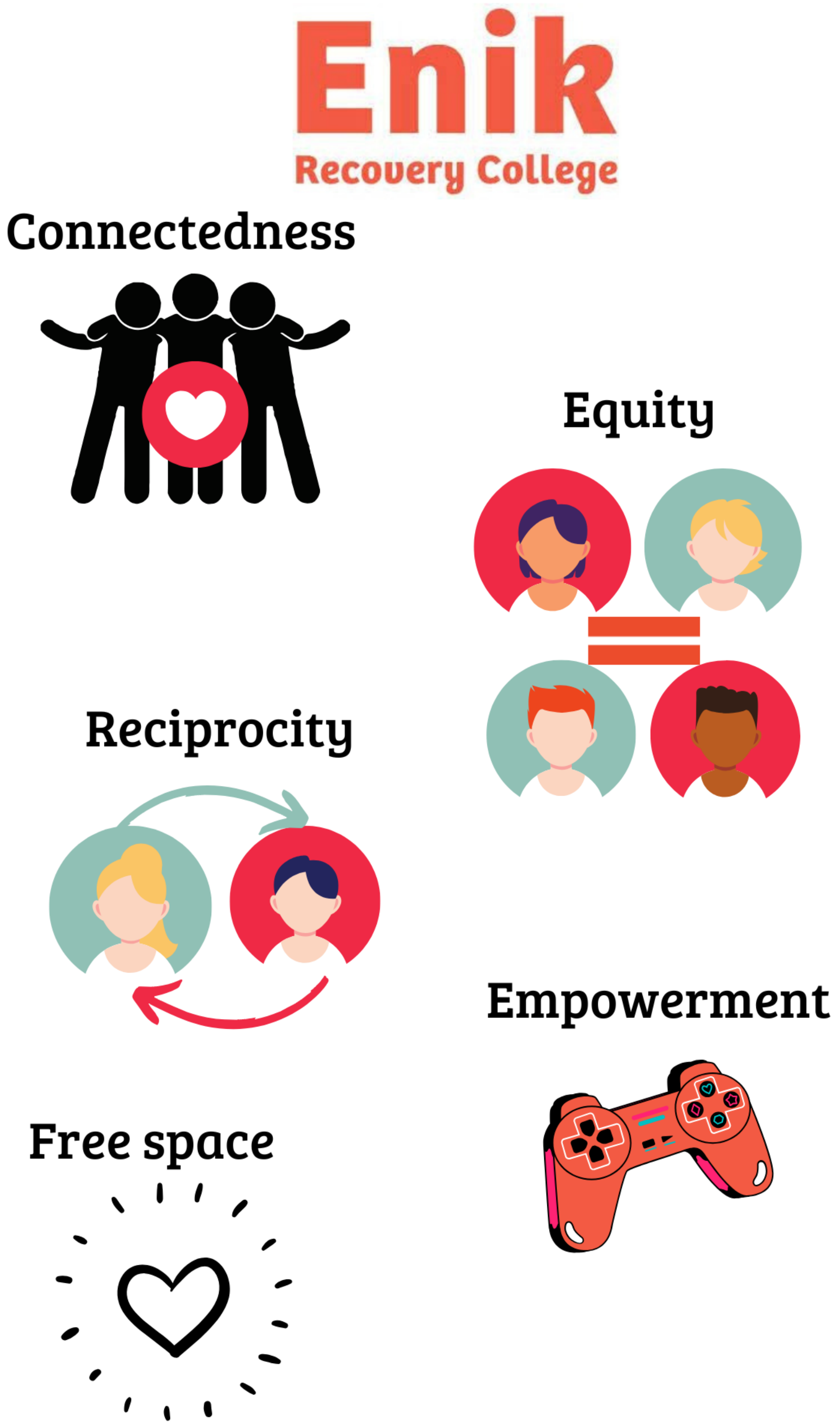
Enik
Recovery College
Connectedness
Equity
Reciprocity
Empowerment
Free space
